# Predicting Subcellular Localization of Apoptosis Proteins Combining GO Features of Homologous Proteins and Distance Weighted KNN Classifier

**DOI:** 10.1155/2016/1793272

**Published:** 2016-04-24

**Authors:** Xiao Wang, Hui Li, Qiuwen Zhang, Rong Wang

**Affiliations:** School of Computer and Communication Engineering, Zhengzhou University of Light Industry, Zhengzhou 450002, China

## Abstract

Apoptosis proteins play a key role in maintaining the stability of organism; the functions of apoptosis proteins are related to their subcellular locations which are used to understand the mechanism of programmed cell death. In this paper, we utilize GO annotation information of apoptosis proteins and their homologous proteins retrieved from GOA database to formulate feature vectors and then combine the distance weighted KNN classification algorithm with them to solve the data imbalance problem existing in CL317 data set to predict subcellular locations of apoptosis proteins. It is found that the number of homologous proteins can affect the overall prediction accuracy. Under the optimal number of homologous proteins, the overall prediction accuracy of our method on CL317 data set reaches 96.8% by Jackknife test. Compared with other existing methods, it shows that our proposed method is very effective and better than others for predicting subcellular localization of apoptosis proteins.

## 1. Introduction

Apoptosis, or programmed cell death, proposed by Professor Kerr in 1972, is the last phase of the cell life, which is the regulatory disintegration of cell. Apoptosis is an important part of many biological processes, such as individual morphogenesis, tissue renewal, neural development, and immune regulation. The proliferation and death of cell can maintain an appropriate number of cells in order to keep balance of biological tissue. If apoptosis malfunctions, diseases such as cancer, AIDS, ischemic damage, and Alzheimer's disease will ensue [1–3]. Knowing the functions of apoptotic proteins helps to understand the mechanism of programmed cell death [4]. Since the functions of the proteins were proved to be closely related to their subcellular locations [5], information about subcellular locations of apoptosis proteins is useful to help us understand the mechanism of apoptosis [6]. The subcellular locations of proteins can be detected by biological experiments, but the method of manual experiment is expensive and time-consuming. With the exponential growth of the number of proteins, proteins which are annotated by biological experiments cannot meet researchers' demand [7]. However, with the help of computer automatic forecasting, we were able to overcome these difficulties.

Many studies have been devoted to developing computational methods to predict the subcellular localization of proteins, and a lot of excellent achievements have been achieved. As pointed in a recent review [8], in the last decade or so, a number of web-servers were developed for predicting the subcellular localization of proteins with both single site and multiple sites based on their sequences information alone. They can be roughly classified into two series [8]. One is the “PLoc” series and the other is the “iLoc” series. The “PLoc” series contains six web-servers [9–14] to deal with eukaryotic, human, plant, Gram-positive, Gram-negative, and virus proteins, while the “iLoc” series contains seven web-servers [15–21] to deal with eukaryotic, human, plant, animal, Gram-positive, Gram-negative, and virus proteins, respectively.

In general, proteins can simultaneously reside at, or move between, two or more subcellular locations. Proteins with multiple locations or dynamic feature of this kind are particularly interesting because they may have some very special biological functions intriguing to investigators in both basic research and drug discovery. So far, many outstanding predictors, that is, iLoc-Euk [18], iLoc-Plant [16], MLPred-Euk [22], MultiP-SChlo [23], mGOASVM [24], and HybridGO-Loc [25], were also developed into web-servers used to cope with the multiple location problems in eukaryotic, plant, virus, and human proteins, respectively. In this study we did not cover the case of multiplex proteins because the number of multiplex proteins in the existing apoptosis protein database is not large enough to construct a statistically meaningful benchmark data set for studying the case of multiple locations.

To develop a computational method for statistically predicting protein subcellular localization, one of the most important steps is to extract core and essential features of protein samples; the approaches can be classified as sequence-based and annotation-based. Sequence-based methods include amino acid compositions [26, 27], sequence homology [28, 29], and sorting signals [30, 31] as features. Annotation-based methods extract information from knowledge databases, such as function domain [32], Gene Ontology [17–19, 24, 25, 33], or Swiss-Prot keywords [34, 35]. Among them, a number of studies of protein subcellular localization prediction have demonstrated that GO annotation methods are superior to methods based on other features [7, 24].

For predicting subcellular localization of apoptosis proteins, in the past 10 years, many studies achieved good results in solving the problem. Since 2003, Doctor and Zhou [36] firstly proposed the study of predicting subcellular localization of apoptosis proteins, built ZD98 data set containing 98 apoptosis proteins with four kinds of subcellular locations, and adopted the covariant discriminant algorithm based on amino acid compositions of the covariant discriminant algorithm. Bulashevska and Eils [37] used Bayesian classifier based on ZD98 data set; in the same year, Zhang et al. [38] combined group weight coding method with support vector machine (EBGW_SVM) on 151 and 225 apoptosis proteins data sets. Chen and Li [39, 40] constructed a new CL317 data set containing 317 apoptosis proteins; the data set had six subcellular locations, using the increment of diversity algorithm (ID) and SVM to predict subcellular locations of apoptosis proteins. Ding and Zhang [41] adopted fuzzy *K*-nearest neighbor algorithm (FKNN) based on pseudo amino acid composition method (PseAAC). Zhang et al. [42] combined distance frequency with SVM, Qiu et al. [43] used wavelet coefficients, and Liu et al. [44] used the autocovariance transformation on position-specific score matrices (PSSM-AC). Lin et al. [45] used PseAAC and SVM. Gu et al. [46] used ensemble classifier and feature selection. Yu et al. [47] used auto covariance transformation based on amino acid substitution matrix. Saravanan and Lakshmi [48] used adaptive boosting classifier. Recently, Zhang et al. [49] used the triplet composition features based on the protein hydropathy characteristics. Liu et al. [50] used trigram encoding based on PSSM.

In previous studies, most of the methods of feature extraction are based on the amino acid or sequences information. These methods are indeed capable of improving the overall accuracy of prediction. However, for the apoptosis proteins, other feature extraction methods, like annotation-based method, especially the Gene Ontology (GO) annotation, were seldom used. According to the former research, the method of GO annotation is proved to be an effective feature extraction method. With the development of GO database, the GO annotation information of apoptosis proteins has become increasingly perfect. In this study, we use the GO annotation information of the apoptosis proteins in CL317 data set and their homologous proteins as the feature extraction method. Considering that the number of proteins contained in each subcellular location is different, some subcellular locations may contain more and some may be less, the so-called class imbalance problems. In order to solve this problem, we select distance weight KNN classification algorithm. Jackknife cross-validation tests on CL317 data set show that our method can achieve higher accuracy than existing methods.

## 2. Materials and Methods

### 2.1. Data Sets

The CL317 data set has 317 apoptosis proteins constructed by Chen and Li [39] that already contain the proteins in ZD98 and ZW225. All proteins in these three data sets are using the same filtering rules from Swiss-Prot. CL317 compared to ZD98 and ZW225 is more innovative and larger. In order to demonstrate the performance of our method, CL317 data set is used in this study. The CL317 data set with six subcellular locations includes 112 cytoplasmic proteins (Cy), 55 membrane proteins (Me), 34 mitochondrial proteins (Mi), 17 secreted proteins (Se), 52 nuclear proteins (Nu), and 47 endoplasmic reticulum proteins (En). With the update and development of the GO database, some of the proteins that are outdated, removed from the database, will not be annotated in the GO database. It means that their Gene Ontology Annotation information will not be retrieved in the GO database. Updating the data set is necessary. In Swiss-Prot (released on 24 July 2015), regarding the two proteins in 112 cytoplasmic proteins, the protein accession numbers (AC) are “P03405” and “Q07814”, but their accession numbers have been turned to “P03404” and “Q07812” which are already included in the cytoplasmic proteins set. The entry of “Q9Z1S4” in nuclear proteins has been removed from the database on 3 November 2009. The total number of data sets is 314 after processing. The number of each class is shown in Table 1.

### 2.2. Gene Ontology Database

It is a problem that the knowledge gotten from different biological databases may be chaotic. The information must be integrated in order to be convenient for biologists. The Gene Ontology (GO) project is to solve the problem and provide consistent descriptors for gene products in different databases. This project first began in 1998 including three databases: Fly Base (Drosophila), the Saccharomyces Genome Database (SGD), and the Mouse Genome Informatics (MGI) project. Since then, the GO Consortium has been developing and expanding, and now it cooperates with many databases of animals, plants, and microbes. GO database is created by the GO Consortium. In the database, GO terms are used to describe characteristics of genes and their products. These are divided into three different types: cellular component, molecular function, and biological process [51].

The Gene Ontology Annotation (GOA) database [52] annotates the genes' products with the definition of GO terms by the GO database and other biological databases. A gene encoding may have a number of different properties, so GO annotation is for the gene product, not the genes. Annotation clarifies the relationship between gene products and the GO terms used to define them. In GOA database, one GO term may be related to many different accession numbers of proteins. Similarly, one AC may correspond to zero or more GO terms. The relationships between ACs and the GO numbers may be many-to-many.

### 2.3. Feature Extraction Methods

Although the GO-based methods have been proved to exhibit excellent performance in the prediction of subcellular locations, there is some controversy or confusion about using this approach. If a protein has already been annotated with the cellular component GO terms, why does one need to predict its subcellular location? Is it merely a procedure of converting the annotation from one format into another? Some facts are shown to illustrate these questions. All the existing benchmark data sets of the existing predictors for protein subcellular localization prediction were established based on the proteins in the Swiss-Prot database, in which their subcellular location information was determined by experiments. Does it mean that outputs from these predictors are not prediction? No, it does not. In fact, for GO and non-GO predictors, by inputting a query protein sequence, without adding any GO information, the output is its subcellular location(s). In other words, as far as the requirement for the input is concerned, there is no difference at all between the non-GO-approach predictors and GO-approach predictors [53]. The good performance of GO-based methods is due to the fact that the features vectors in the GO space can better reflect their subcellular locations than those in the Euclidean space or any other simple geometric space [54]. And our previous work [33] also strongly supports the legitimacy of using GO information for subcellular localization prediction. Other studies [24, 55] have demonstrated that solving the prediction problem by creating a lookup table using the cellular component GO terms and the cellular component categories is not desirable and has very poor prediction performance.

According to our previous work [33, 56], we first compress and reorganize the GO numbers in GO database (released on 20 June 2015), because the GO number is not continuous. We map GO numbers to GO_compress numbers and create a new database called GO_compress database. The new database is used to store the data after processing.

As time goes on, the number of GO terms is increasing rapidly. It is impossible to use all of the GO terms used to generate the feature vector; otherwise, it will face high dimensional data disaster. In this study, GO terms marked “cell component” in GO database are selected, which contains 3951 GO numbers. We deal with these GO numbers using the above methods.

The protein P is represented as

(1) $$\begin{array}{c} P={\left[\;\begin{array}{ccccccc} f_{1} & f_{2} & f_{3} & \cdots & f_{u} & \cdots & f_{3951} \end{array}\;\right]}^{T}, \end{array}$$

where *f*_*u*_ are defined as follows.

BLAST was used to search the Swiss-Prot (released on 24 July 2015) and find the homologous proteins of P and these homologous proteins are collected into a set. The proteins in the set are seen as “representative proteins” of P, sharing some similar attributes such as structural conformations and biological functions.

If the set is null, that is, P has no homologous proteins, or homologous proteins have no GO numbers, only use the P itself to search the GO database, find the corresponding GO number(s), and then convert the GO numbers to their GO_compress numbers. We have mentioned that an AC of protein in Uniprot/Swiss-Prot may correspond to 0, 1, or more GO number(s); the relationship between AC and the GO numbers may be one-to-many. If the set is not null, use the P and the homologous proteins in the set to search the GO database, find the corresponding GO number(s), and then convert the GO numbers to their GO_compress numbers. We find that the results of predicting are different with using different number of homologous proteins in the set. We will conduct a detailed description in the following.

*f*_*u*_ is defined as

(2) $$\begin{array}{c} f_{u}=\frac{\sum_{j=1}^{N_{P}^{h}}\theta \left(u,j\right)}{N_{P}^{h}}\;\left(u=1,2,3,\ldots ,3951\right), \end{array}$$

where *N*_P_^*h*^ is the number of P and the homologies in the set; if *j*th representative protein hits the *u*th GO_compress number, then *θ*(*u*, *j*) = 1; otherwise, *θ*(*u*, *j*) = 0. All proteins in the data set have been annotated by GO database; GO numbers of proteins can be found in GOA database; it will not appear that the feature vector created by using this method is naught vector under the condition that the number of the homologous proteins is 0.

### 2.4. Distance Weighted KNN Classification Algorithm

*K*-nearest neighbor classification algorithm is as follows: when a test sample (unknown sample) is given, firstly search the pattern space to find out the *K* training samples (known samples) which are closest to the test samples, namely, *K*-nearest neighbors, and then count the selected *K*-nearest neighbors; if a class has the largest number of the nearest neighbors, the test sample is determined to belong to the class. Euclidean distance is used to calculate the distance between the test sample and all the training samples. The formula is

(3) $$\begin{array}{c} \text{distance}\left(\;X,Y\;\right)=\sqrt{\overset{N}{\underset{i=1}{\sum }}{\left(x_{i}-y_{i}\right)}^{2}}, \end{array}$$

where *X* is a test sample and *Y* is a training sample.

However, the algorithm has a very obvious deficiency; when the number of samples is not balanced, such as one class having a large number of samples, while the other classes are small, it may be the case of classification errors, because in the prediction of new samples, the most *K* neighbors belong to the large capacity classes. In this study, the classification of samples in Mi and Se may be mispredicted. In order to solve the data imbalance problem, we use the distance weight KNN classification algorithm. The weight is equal to the reciprocal of the distance between the two samples. Consider

(4) $$\begin{array}{c} weight=\frac{1}{\text{distance}\left(X,Y\right)}. \end{array}$$

The smaller the distance, the greater the weight. For a test sample, find *K*-nearest neighbors, calculate the weights, and add together the weights of the samples belonging to the same classes; the class of the test sample is the highest value one.

### 2.5. Prediction Process

**Figure 1:**
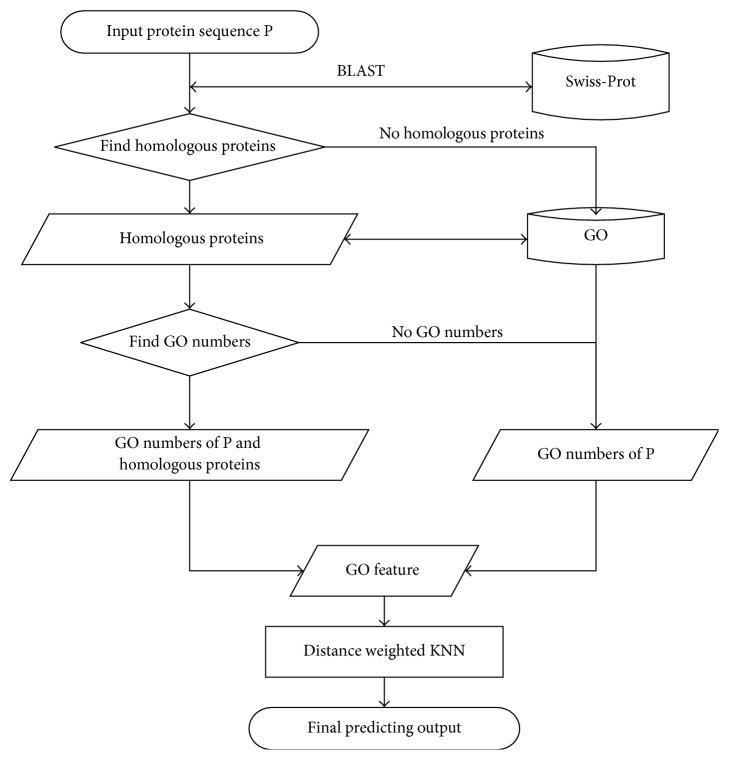
A flowchart to show the prediction process.

Input a protein sequences P, first use the BLAST to search the Swiss-Prot database to find the homologous proteins of P and collect these proteins into a set, and then search GO database to find the GO numbers of the P and its homologous proteins. If the set is null or these homologous proteins have no GO numbers, only use P itself to search GO database. Input the GO features formulated by GO numbers to the distance weighted KNN classifier and get the result of predicting. To provide an intuitive picture, a flowchart is provided in Figure 1 to illustrate the prediction process.

### 2.6. Performance Measures

In statistical prediction, for objectively evaluating performance or anticipated success rate, independent inspection, *k*-fold cross test, and Jackknife test are three common testing methods, where the Jackknife test is the most rigorous and objective testing method. In the Jackknife test, the data is divided into *N* subsets; that is, each subset is as a test set, and the remaining *N* − 1 proteins are as a training set, cycle *N* times, and each extracted sample should be put back to the data set. In this paper, we use Jackknife test.

Four standard performance measures, sensitivity (SN), specificity (SP), Matthews correlation coefficient (MCC), and the overall accuracy (ACC), used in [57–62] were adopted. The definitions are shown as follows:

(5) ACC=∑i=1MN+i−N−+iN,SNi=1−N−+iN+i,SPi=1−N+−iN−i,MCCi=1−N−+i/N+i+N+−i/N−i1+N+−i−N−+i/N+i1+N−+i−N+−i/N−i,

where *i*  (*i* = 1,2,…, 5, 6) is subcellular subset, *N* ^+^(*i*) is the total number of the apoptosis protein sequences in subset *i*, and *N*_−_^+^(*i*) is the number of apoptosis protein sequences in *i* incorrectly predicted to belong to the other subsets; and *N* ^−^(*i*) is the total number of the apoptosis protein sequences in all of the other subsets and *N*_+_^−^(*i*) is the number of the apoptosis protein sequences incorrectly predicted to belong to *i*.

Obviously, when *N*_−_^+^(*i*) = 0, meaning that none of the apoptosis protein samples in subset *i* was incorrectly predicted to belong to other subsets, SN_*i*_ = 1; when *N*_−_^+^(*i*) = *N* ^+^(*i*), meaning that all samples in *i* were incorrectly predicted to belong to the other subsets, SN_*i*_ = 0. Likewise, when *N*_+_^−^(*i*) = 0, meaning that none of the protein samples in the other subsets was incorrectly predicted to belong to the subset *i*, SP_*i*_ = 1; when *N*_+_^−^(*i*) = *N* ^−^(*i*), meaning that all samples in the other subsets were incorrectly predicted to belong to *i*, SP_*i*_ = 0. When *N*_−_^+^(*i*) = *N*_+_^−^(*i*) = 0  (*i* = 1,2,…, 5,6), meaning that all samples in the subsets were correctly predicted, ACC = 1; when *N*_−_^+^(*i*) = *N* ^+^(*i*) and *N*_+_^−^(*i*) = *N* ^−^(*i*)  (*i* = 1,2,…, 5,6), meaning that none of samples in all subsets was correctly predicted, ACC = 0. The MCC is usually used for measuring the quality of binary (two-class) classifications. When *N*_−_^+^(*i*) = *N*_+_^−^(*i*) = 0, meaning that all samples in *i* were correctly predicted, MCC_*i*_ = 1; when *N*_−_^+^(*i*) = *N* ^+^(*i*)/2 and *N*_+_^−^(*i*) = *N* ^−^(*i*)/2, MCC_*i*_ = 0, meaning no better than random prediction for samples in *i*. When *N*_−_^+^(*i*) = *N* ^+^(*i*) and *N*_+_^−^(*i*) = *N* ^−^(*i*), MCC_*i*_ = −1, meaning total disagreement between prediction and observation for samples in *i*. As we can see from the above, it is much more intuitive and easier-to-understand for four metrics when evaluating the performance of the predictor, particularly for its Matthews correlation coefficient.

It should be pointed out that the set of metrics defined in (5) is valid only for the single-label systems. For the multilabel systems whose existence has become more frequent in system biology [19] and system medicine [63], a completely different set of metrics as defined in [5, 53] is needed.

## 3. Results and Discussion

### 3.1. Effect of the Number of Homologous Proteins

**Figure 2:**
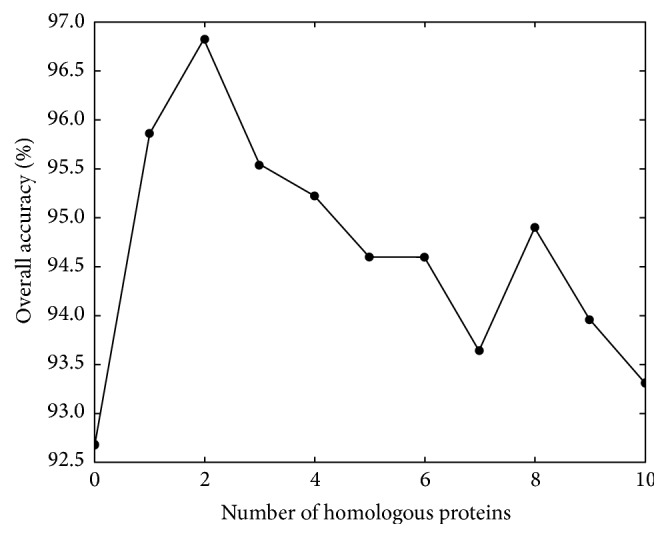
This graph shows how different numbers of homologous proteins affect the overall accuracies.

Through experiment, it is found that the overall prediction accuracy is changed with the increasing of the number of the homologous proteins. We select from 0 to 10 of the homologous proteins of one protein. The order of homologous proteins is done according to the sequence similarity; the homology used first is the highest similarity. If a protein does not have so many homologous proteins, we select all of its proteins, and if a homology has no GO numbers, we will ignore it.  Figure 2 shows the details. In Figure 2, the horizontal coordinates represent the number of homologous proteins, and the longitudinal coordinates represent the overall prediction accuracy. As can be observed from the figure, the number is 0 and the overall accuracy is 92.7%; the number is 1, the overall accuracy is 95.9%, and so forth. The effect of the addition of homology is better than that using only the protein itself. When the number is less than or equal to 2, the accuracy shows a rising trend; when the number is 2, the highest accuracy is reached; with the increase of the number, the prediction accuracy is decreased. The reason may be that the GO information contained in the homologous proteins is more than that in itself. With increasing of the number, the GO feature information will be more abundant. However, too much information will become redundant information and will reduce the accuracy.

### 3.2. Prediction Performances of Our Method

By Jackknife test, our method is examined with updated CL317 data set, selecting 2 homologous proteins and reporting SN, SP, and MCC for each subcellular location, as well as ACC. The results are shown in Table 2.

### 3.3. Performance Comparison with Existing Methods

In order to further evaluate the performance of current method objectively, we compare the other methods using CL317 data sets by Jackknife test; the results are shown in Table 3. Table 3 shows that the ACC of our method is 96.8%, better than any other method. Predicted results on Me, Mi, Se, and En subcellular locations are higher than other methods. Our method can achieve good classification results in small samples such as Mi and Se. This illustrates that our approach can do well when dealing with data imbalance. But it is noticed that the results of Cy and Nu are not so good, lower than APSLAP and trigram encoding. It may be due to the small number of homologous proteins in the two classes, or homologous proteins have less GO numbers. For a protein, relatively abundant GO annotation information can improve the accuracy of prediction. In conclusion, the outstanding performance can be ascribed to the effective usage of feature extraction method based on GO annotations of homologous proteins and distance weighted KNN classification algorithm.

## 4. Conclusions

In previous studies, most of the feature extraction methods are based on the amino acid sequence. Using the annotation methods, especially GO annotation, is less in this research. Because the GO annotation information of a protein is very limited, we use the GO information of itself and its homologies to express the features of a protein. We first obtain the homologous proteins of proteins, search GO database using them to find the GO numbers, and then formulate the feature vectors. Finally, the feature vectors are selected to perform the prediction by distance weighted KNN classifier. While the number of homologous proteins is set to 2, the prediction accuracy on the CL317 data set by Jackknife test reaches 96.8%, outperforming other existing methods. The experimental results show that our method provides the state-of-the-art performance for predicting subcellular localization of apoptosis proteins. Our next job will be to provide a better solution to this problem. To provide prediction service for more researchers, here we have provided a web-server for the method presented in this paper at http://biomed.zzuli.edu.cn/bioinfo/apoptosis/.

## Figures and Tables

**Table 1:** Number of proteins in each of the 6 subcellular locations.

Subset	Subcellular location	Number of proteins
1	Cytoplasmic	110
2	Membrane	55
3	Mitochondrial	34
4	Secreted	17
5	Nuclear	51
6	Endoplasmic reticulum	47
Total number		314

**Table 2:** The prediction result for the data set.

Location	SN (%)	SP (%)	MCC
Cy	98.2	97.5	0.951
Me	98.2	99.6	0.978
Mi	97.1	99.3	0.951
Se	94.1	100	0.968
Nu	90.2	99.2	0.917
En	100	100	1.0
ACC	96.8		

**Table 3:** Comparison of different methods on CL317 data set.

Method	SN (%)						ACC (%)
	Cy	Me	Mi	Se	Nu	En	
ID [39]	81.3	81.8	85.3	88.2	82.7	83.0	82.7
ID_SVM [40]	91.1	89.1	79.4	58.8	73.1	87.2	84.2
DF_SVM [42]	92.9	85.5	76.5	76.5	93.6	86.5	88.0
Auto_Cova [47]	86.4	90.7	93.8	85.7	92.1	93.8	90.0
FKNN [41]	93.8	92.7	82.4	76.5	90.4	93.6	90.9
PseAAC_SVM [45]	93.8	90.9	85.3	76.5	90.4	95.7	91.1
EN_FKNN [46]	98.2	83.6	79.4	82.4	90.4	97.9	91.5
PSSM-AC [44]	93.8	90.9	91.2	82.4	86.5	95.7	91.5
APSLAP [48]	99.1	89.1	85.3	88.2	84.3	95.8	92.4
Trigram encoding [50]	98.2	96.4	94.1	82.4	96.2	95.7	95.9
Our method	98.2	98.2	97.1	94.1	90.2	100	96.8
